# Unique Presentation of Septic Cavernous Sinus Thrombosis and Pulmonary Embolism in the Setting of Reusable Face Covering

**DOI:** 10.1155/2022/3388537

**Published:** 2022-04-15

**Authors:** Javier Barranco-Trabi, Jazmin C. Scott, Jacob M. Fryer, Matthew Byrne, Adam Smith, Kaoru H. Song, Chelsea W. Tagawa, Sharon Chi, Viseth Ngauy

**Affiliations:** ^1^Department of Medicine, Internal Medicine, Tripler Army Medical Center, Honolulu, HI 96859, USA; ^2^Department of Psychiatry, Tripler Army Medical Center, Honolulu, HI 96859, USA; ^3^Uniformed Services University of the Health Sciences, Bethesda, MD 20814, USA; ^4^Department of Radiology, Tripler Army Medical Center, Honolulu, HI 96859, USA; ^5^Department of Family Medicine, Tripler Army Medical Center, Honolulu, HI 96859, USA; ^6^Infectious Diseases, Veterans Affairs Pacific Islands Health Care System, Honolulu, HI 96819, USA; ^7^Division of Infectious Disease, Tripler Army Medical Center, Honolulu, HI 96859, USA

## Abstract

Identified in December 2019, SARS-CoV-2 quickly spread worldwide with a resultant increase in global morbidity, mortality, and economic disruption on a scale not seen since the 1918 Spanish flu. Health officials recommended universal masking to further reduce human-to-human spread of SARS-CoV-2. The state of Hawaii and the Department of Defense (DOD) adopted strict mask policies early in the pandemic and is shown to be effective at reducing transmission. We report a case of *Staphylococcus aureus* bacteremia in an immunocompetent 21-year-old man attributed to local skin irritation with resultant infection in the setting of continuous reuse of a mask that resulted in bilateral cavernous venous thrombosis and septic pulmonary embolism.

## 1. Introduction

During the early stages of the SARS-CoV-2 pandemic following initial reports from Wuhan, China, in December of 2019, transmission, potential for airborne transmission via aerosols, and infectiousness of asymptomatic were not clear. It was not until April 2020 that the Centers for Disease Control and Prevention (CDC) incorporated recommendations for mask wearing in public settings to prevent respiratory transmission. The state of Hawaii and the Department of Defense (DOD) adopted strict policy in accordance with the CDC guidelines, mandating universal masking in public settings. Cloth face coverings were considered an acceptable alternative to surgical and N95 masks for general public use to offset the shortages of personal protective equipment in healthcare settings. Acute bacterial skin and skin-structure infection (ABSSSI) secondary to mask wearing has not been previously described in the literature, and to the best of our knowledge, we report the first case of a septic cavernous sinus thrombosis and pulmonary embolism arising from complications of poor hygiene with reusable face coverings.

Multiple factors can lead to acute skin infections of the face including imbalance of the skin's microbiota [1], trauma, irritation, inflammation, and structural impediments. The most common bacterial pathogens are staphylococcal and streptococcal species [2]. *Staphylococcus aureus* can be found in anterior nares in up to 20–30% of a population [1, 2] with higher rates observed among those with certain conditions such as HIV, diabetes, those on dialysis, and among persons who inject drugs (IDU) [3, 4]. Mortality associated with *S. aureus* bacteremia is 20–40% [4].

Infections involving the “danger triangle,” an area defined by both corners of the mouth to the bridge of the nose, may lead to cavernous sinus thrombosis (CST). Cavernous sinus thrombosis is a life-threatening disorder that can result in further complications from propagation of thrombosis deeper in the venous drainage of the cavernous sinus to the pterygoid plexus, angular veins, and ophthalmic veins. This anatomy increases the possibility of an infection spreading intracranially resulting into septic CST [1, 5]. Cavernous sinus thrombosis comprises approximately 1% to 4% of all cerebral venous and sinus thrombosis (CVST), which has an annual incidence of approximately two to four per million people per year. The annual incidence of cavernous sinus thrombosis may be approximately 0.2 to 1.6 per 100,000 per year [4, 5].

## 2. Case Presentation

**Figure 1:**
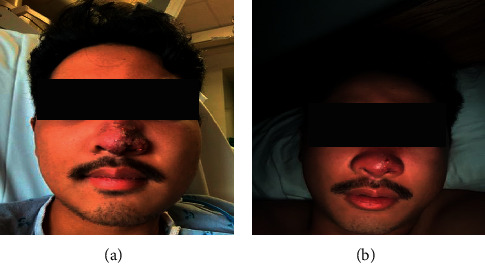
(a) Presentation of the patient in the Emergency Room. (b) One week of antimicrobial therapy.

A 21-year-old active-duty male in the U.S. Navy presented with new onset facial swelling and painful rash on the left side of his nose starting three days prior. He described first noticing 2-3 “pimples” on his nose (see Figure 1). After picking them, the lesions increased in number [4–6], and swelling worsened followed by headache that progressively worsened. This was accompanied by further nasal swelling, pain with eye movements, nausea, anorexia, photophobia, neck stiffness, shortness of breath, and subjective fevers. The patient denied any past medical history. He was fully vaccinated with two doses of the Pfizer BioNtech SARS-CoV-2 vaccine. His occupation involved shipyard work with daily patrol duties. Wearing of a mask or face covering was required while on duty and in the general public during this time. He was a previous smoker of cigarettes but recently switched to daily vaping. The patient reported owning only one cloth mask, but had not laundered his masks for over a month.

**Figure 2:**
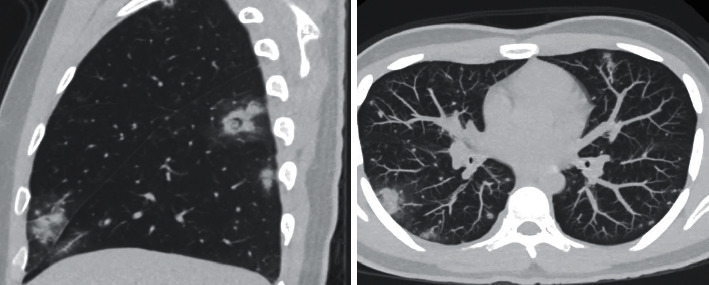
Sagittal and axial MIP CT reconstructions demonstrate numerous round centrilobular nodules, with the larger nodules demonstrating areas of central necrosis and peripheral ground glass opacity.

On presentation, he had a blood pressure of 150/78 mmHg and temperature of 102° F. On physical exam, the patient had significant facial edema involving the lips and eyelids and conjunctivitis with slight discharge from both eyes. Extraocular movements were intact with mild discomfort, without diplopia. Nasal discharge with serosanguinous fluid, cervical lymphadenopathy with mild tenderness to palpation, seven 1-2 mm pustules on the left ala and apex of his nose with surrounding erythema and edema, and nuchal rigidity were present. Labs were notable for a white blood cell count of 28.53 × 10^3/ul, hematocrit 34.4%, procalcitonin of 3.47 ug/L, D-dimer of 6.21 ug/mL, C-reactive protein of 142.8 mg/L, erythrocyte sedimentation rate of 29 mm/hr, and platelet count of 501 × 10^9. SARS-CoV-2 PCR on admission was negative. Computed tomography of the head with contrast showed evidence of cellulitis along the inferior frontal scalp, periorbital regions, and face without abscess, and mildly enlarged cervical chain lymph nodes with filling defect located centrally within the IJV at the level of C1. Computed tomography of the chest increased suspicion for septic emboli (see Figure 2). Broad-spectrum antimicrobial therapy was initiated with vancomycin and piperacillin/tazobactam.

Two sets of blood cultures were positive within 24 hours for methicillin sensitive *Staphylococcus aureus* (MSSA). Antimicrobial therapy was adjusted to intravenous oxacillin via continuous infusion and gentamicin. Blood cultures remained positive for 2 additional days.

**Figure 3:**
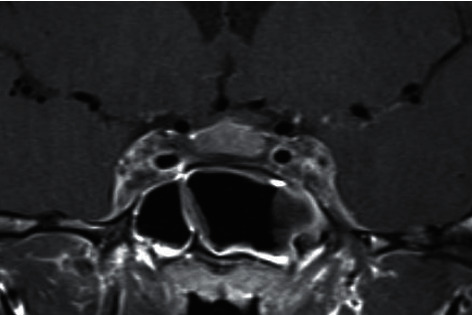
Coronal T1 postcontrast MRI demonstrates an enlarged and rounded appearance of the cavernous sinus with heterogeneous enhancement, consistent with cavernous sinus thrombosis.

There was no evidence of endocarditis on transthoracic and transesophageal echocardiograms. Further imaging included a head CT venogram which showed convexity of bilateral cavernous sinuses with filling defects indicative of bilateral cavernous sinus thromboses. Coronal T1 postcontrast MRI demonstrated an enlarged and rounded appearance of the cavernous sinus with heterogeneous enhancement, consistent with cavernous sinus thrombosis (see Figure 3). MRI of the sella turcica also showed a filling defect within the left internal jugular vein (IJV) from the jugular foramen to the level of C1 with preservation of flow distal and proximal, suspicious for nonocclusive thrombosis.

In regard to anticoagulation in cavernous sinus thrombosis, given a known left IJV venous thrombus with septic emboli and the risk of propagation of that thrombus, we decided to anticoagulate with a heparin drip with no bolus. The patient was then transitioned to enoxaparin for a seven-day treatment course.

During his hospital course, he developed a mild hypertransaminasemia, which prompted switching from oxacillin to cefazolin. He completed 6 weeks of this treatment, with improvement at follow-up.

## 3. Discussion

*Staphylococcus aureus* bacteremia with dissemination to end organs can be a deadly consequence of local ABSSSI. The anterior nares are common sites of *S. aureus* colonization. The development cellulitis from local irritation or trauma in the facial area can lead to CNS infection either by direct extension or via seeding of the blood stream. Our patient ultimately developed *S. aureus* bacteremia with cavernous sinus and pulmonary thrombotic complications from local skin irritation related to continuous prolonged reuse of an unlaundered face covering. He wore his mask for many hours during the day in a humid environment with nonadherence to reusable cloth mask hygiene guidance provided by the CDC. “Maskne” refers to acne breakouts or flares as a result of mask use [6]. While this is a rare and extremely severe case of infection that may have started as “maskne,” it highlights the importance of maintaining good mask hygiene with regular laundering of reusable masks, or use of disposable masks to avoid development of “maskne” in the danger triangle. The ongoing use of face coverings and masks is not expected to stop in the near future with reports of more transmissible circulating SARS-CoV-2 across the globe [7]. Furthermore, certain individuals who are at high risk for complications of SARS-CoV-2 infection including those who are immunocompromised may continue to use masks even as wider mask mandates are lifted in the United States. As people continue to use face coverings, it is important to emphasize proper care and cleaning of face coverings and reusable masks to prevent these adverse outcomes.

The presence of a cavernous sinus thrombosis in our patient was an unexpected finding. Cavernous sinus thrombosis is a feared complication of facial or oropharyngeal infections such as sinusitis or facial cellulitis [8–10]. Bacteria such as *S. aureus* and streptococcal species are the most common culprits. In septic cavernous sinus thrombosis, antibiotics are undoubtedly beneficial, but additional treatment with anticoagulants has remained controversial [3, 8, 9]. Some studies strongly recommend anticoagulation for improved mortality, but duration of treatment and choice of anticoagulant are still not determined.

## 4. Conclusion

The COVID-19 pandemic has affected many aspects of our daily lives, with guidelines continuously being updated based on best practices and emerging data. Current guidelines recommend the use of reusable or disposable masks in public indoor areas to help decrease the risk of disease transmission. Additionally, at the time this case report was written, the state of Hawaii, where the patient currently resides, required face masks to be worn in public areas. The wearing of face masks during the COVID-19 pandemic is an extremely low-risk public health intervention but is not entirely risk free as evidenced by this patient. Improper or lack of cleaning of reusable face coverings and masks as well as the reuse of disposable face masks may increase the risk of facial skin infections and their complications.

It is important to educate the public on best practices for the hygiene of face masks to decrease the risk of skin infection. These practices include regular laundering of reusable face coverings with attention in replacing with a clean or new mask at least daily and when soiled. Disposing of single use surgical masks with avoidance of continuous use is also noted. Guidance for overall facial hygiene such as minimizing touching, scratching, and picking of facial skin could help preserve the natural skin barrier and limit traumatic points of entry for pathogens. Use of face masks have decreased COVID-19 transmission, but this case serves as a strong reminder that proper hygiene should be included in public health guidelines.

## Data Availability

No data were used to support this study.
